# *HLF* is a promising prognostic, immunological, and therapeutic biomarker in human tumors

**DOI:** 10.1016/j.bbrep.2024.101725

**Published:** 2024-05-01

**Authors:** Mohsen Ahmadi, Amirhossein Mohajeri Khorasani, Firouzeh Morshedzadeh, Negin Saffarzadeh, Sayyed Mohammad Hossein Ghaderian, Soudeh Ghafouri-Fard, Pegah Mousavi

**Affiliations:** a Department of Medical Genetics, Faculty of Medicine, Hormozgan University of Medical Sciences, Bandar Abbas, Iran; b Department of Medical Genetics, School of Medicine, Shahid Beheshti University of Medical Sciences, Tehran, Iran; c Department of Genetics, Faculty of Basic Sciences, Shahrekord Branch, Islamic Azad University, Shahrekord, Iran; d Department of Medical Genetics and Molecular Medicine, Faculty of Medicine, Mashhad University of Medical Sciences, Mashhad, Iran; e Department of Medical Genetics, School of Medicine, Tehran University of Medical Sciences, Tehran, Iran; f Molecular Medicine Research Center, Hormozgan Health Institute, Hormozgan University of Medical Sciences, Bandar Abbas, Iran

**Keywords:** Bioinformatics, Biomarkers, Tumor-infiltrating immune cells, Prognosis, Target therapy, Cancer genetics

## Abstract

Despite past research linking *HLF* mutations to cancer development, no pan-cancer analyses of *HLF* have been published. As a result, we utilized multiple databases to illustrate the potential roles of *HLF* in diverse types of cancers. Several databases were used to assess *HLF* expression in the TCGA cancer samples. Additional assessments were undertaken to investigate the relationship between *HLF* and overall survival, immune cell infiltration, genetic alterations, promoter methylation, and protein-protein interaction. HLF's putative roles and the relationship between *HLF* expression and drug reactivity were investigated. *HLF* expression was shown to be lower in tumor tissues from a variety of malignancies when compared to normal tissues. There was a substantial link found between *HLF* expression and patient survival, genetic mutations, and immunological infiltration. HLF influenced the pathways of apoptosis, cell cycle, EMT, and PI3K/AKT signaling. Abnormal expression of *HLF* lowered sensitivity to numerous anti-tumor drugs and small compounds. According to our findings, reduced *HLF* expression drives cancer growth, and it has the potential to be identified as a vital biomarker for use in prognosis, immunotherapy, and targeted treatment of a range of malignancies.

## Highlights

• HLF expression was lower in tumor tissues when compared to normal tissues.
• There was a link between HLF expression and patient survival, mutations, and immunological infiltration.
• HLF influenced the pathways of apoptosis, cell cycle, EMT, and PI3K/AKT signaling.
• Abnormal expression of HLF lowered sensitivity to numerous anti-tumor drugs and small compounds.
• HLF has the potential to be a biomarker for prognosis, immunotherapy, and targeted treatment.

## 1 Introduction

Cancer is a serious hazard to public health owing to substantial rise in global warming and poor lifestyles [1]. The pathophysiology of cancer is exceedingly complicated, making early detection challenging. Furthermore, established diagnostic and therapeutic procedures are ineffective in the early stages of diagnosis and treatment [[2], [3], [4]]. Developments in sequencing technology and online datasets have allowed us to understand some genes' roles in human cancer [5,6]. For example, The TCGA project has information on 33 malignancies and over 10,000 individuals [7]. Therefore, we can now better understand how particular genes function in human cancer.

Circadian disruption has been associated with elevated susceptibility to malignancies such as breast, prostate, colorectal, liver, and non-Hodgkin's lymphoma in recent research [[8], [9], [10], [11], [12]]. In point of fact, there is substantial evidence suggesting that shift work, which disrupts humans' natural circadian rhythms, is detrimental to their health. A complex auto-regulatory network of 'clock' genes is responsible for orchestrating circadian rhythms. These genes govern physiological and behavioral functions in response to periodic changes in the environment. Hepatic leukemia factor, often known as *HLF*, is a clock-dependent transcription factor that plays an important role in the circadian adjustment of a number of different activities [[13], [14], [15]]. It belongs to the proline- and acidic amino acid-rich basic leucine zipper protein family and was initially found in early B-lineage acute leukemia patients who had aberrant expression of the transcription factor E2–HLF fusion gene. Subsequently, it was discovered to be expressed in liver and kidney cells [16,17]. Furthermore, the *HLF* gene has been uncovered to have a crucial regulatory function in developing several malignancies, including lung, renal, glioma, liver, and breast [[18], [19], [20], [21], [22], [23]]. These findings raise the idea that *the HLF* gene plays diverse functions during tumorigenesis depending on the tissue and setting. However, it is still unclear whether the *HLF* gene is implicated in the etiology of cancers and could be regarded as a possible target and a crucial mediator in a number of human cancers via a shared signaling mechanism.

We utilized the data from the TCGA dataset to perform a pan-cancer analysis of the *HLF* gene in this research. To unravel the molecular processes of the *HLF* gene in cancer, we explored the association of *HLF* expression with prognosis, genetic changes, the immunological microenvironment, gene function, and drug sensitivity of cancer patients.

## 2 Materials and methods

### 2.1 Gene expression analysis

We applied the “TCGA” option of the UALCAN platform to conduct a comparison of the levels of *HLF* expression in tumor tissues and normal samples across 24 human cancers in the TCGA project. UALCAN (http://ualcan.path.uab.edu/analysis.html) is a powerful web tool that allows access to available cancer omics data [24,25]. For those cancers with insufficient normal sample size or with highly limited normal tissues (N < 10), or without available data in the UALCAN database, we utilized the “box plot” tab of “Expression DIY” module of the GEPIA2 web server to assess the expression difference between the tumor tissues of TCGA cohort and the related normal tissues of the TCGA and the GTEx projects, with predefined settings (q-value cutoff = 0.01, log2FC (Fold Change) cutoff = 1). The GEPIA2 (http://gepia2.cancer-pku.cn/#analysis) is an online TCGA data analysis tool that enables researchers to perform various analyses such as expression analysis and survival analysis for a given gene [26]. Cholangiocarcinoma, Sarcoma, Pheochromocytoma and Paraganglioma, Mesothelioma, and Uveal Melanoma were excluded from our analysis because of limited normal tissues (N < 10) in both databases. Furthermore, we analyzed the difference in the *HLF* gene expression between pathological stages of human cancers by the GSCA database (http://bioinfo.life.hust.edu.cn/GSCA/#/). The GSCA platform is a web tool that combines omics data retrieved from on TCGA database. It enables researchers to perform several analyses, including expression analysis, pathway activity, and drug sensitivity [27]. Table S1 shows the abbreviation and sample size for 33 cancer types deposited in the TCGA and GTEx datasets according to GEPIA2 database.

### 2.2 Survival prognosis analysis

We employed the “Survival Map” module of the GEPIA2 database and the "pan-cancer” option of the km-plotter database to investigate the OS of cancer patients with aberrant expression of the *HLF* gene. We also evaluated the association between dysregulation of the *HLF* gene and OS outcome across different tumors through the GSCA and the Kaplan Meier plotter (KM-plotter) databases. The Kaplan-Meier (https://kmplot.com/analysis/) is an online resource that can explore the influence of 54,000 genes on survival outcomes in 21 carcinoma types [28]. These assessments were performed under the following conditions: Based on the median expression levels of the *HLF* gene, cancer patients were divided into high-expression (Cutoff-high (50 %)) and low-expression (cutoff-low (50 %)) groups. A log-Rank p-value <0.05 was considered statically significant.

### 2.3 Genetic alteration analysis

The cBioPortal database (https://www.cbioportal.org/) is an open resource for cancer genomics, providing easy access to data including copy number changes, aberrations in mRNA expression, DNA methylation, and protein expression for more than 5000 tumor samples among more than 20 carcinoma studies [29]. Utilizing the cBioPortal website, we employed the "TCGA Pan-Cancer Atlas Studies" option to explore genetic aberrations of the *HLF* gene. The “Mutations” module also was applied to investigate the mutation site information for the *HLF* gene.

### 2.4 DNA methylation analysis

Using data from the DNMIVD (http://1193.41.228/dnmivd/) database [30], we determined the methylation status of the *HLF* gene in all accessible TCGA cohorts. For comparing the methylation levels between cancer and normal samples, an independent Student's t-test was conducted, and cancers with |beta difference|>0.1 and independent Student's t-test adjusted p-value <0.05 were considered tumors with significant changes in the promoter of the *HLF* gene. In addition, whenever we found the promoter of the *HLF* gene was abnormally methylated, we also explored the association between gene expression and promoter methylation in primary tissues using the Pearson and Spearman correlation analysis with predefined criteria (rho value < -1 and p-value <0.05).

### 2.5 Protein-protein interaction (PPI) analysis

We used the GeneMANIA (http://www.genemania.org) web tool [31] to construct PPI network, including physical interaction, co-localization, prediction, co-expression, shared protein domains, and genetic interaction connections between the *HLF* gene and related genes.

### 2.6 Pathway Enrichment analysis

Utilizing the GSCA database, we investigated the association between the expression of the *HLF* gene and pathway activity across all TCGA tumors. The pathway GSCA contains TSC/mTOR, Receptor Tyrosine Kinase (RTK), RAS/MAPK, PI3K/AKT, Hormone ER, Hormone AR, EMT, DNA Damage Response, Cell Cycle, Apoptosis pathways which recognized as famous cancer-related pathways. In this analysis, on the basis of the median *HLF* gene expression, samples were separated into two groups (High and Low). The Student's T-test calculated the difference in PAS. Then p-value was adjusted by the FDR method; FDR ≤ 0.05 is recognized as statistically significant. When PAS for samples with High expression of the *HLF* gene was greater than PAS of samples with Low expression, we supposed that the *HLF* gene might promote pathway activity, otherwise suppressing pathway function.

### 2.7 Immune infiltration analysis

We utilized the “Immune-Gene” module of the TIMER2 (http://timer.comp-genomics.org/) database [32] to examine the correlation between the *HLF* gene expression and immune infiltrates among all available TCGA cohorts. The CIBERSORT-ABS method was used to conduct this assessment, and the p-value and partial correlation (cor) values were corrected for tumor purity using Spearman's rank correlation test. A p-value <0.05 was considered statistically significant. Table S2 indicates the immune cells selected for our analysis.

### 2.8 Drug sensitivity analysis

We used the GSCA database to conduct drug sensitivity analysis to discover whether abnormal expression of the *HLF* gene affects cancer patients' clinical response and targeted therapy. This platform has gathered the IC50 of 265 small molecules in 860 cell lines and related mRNA gene expression from the GDSC. It conducts Spearman's correlation analysis to assess the correlation between the expression of a given gene with drug sensitivity. The positive association unveils that the overexpression of a gene may be related to drug resistance and vice versa.

## 3 Results

### 3.1 Expression analysis

**Fig. 1:**
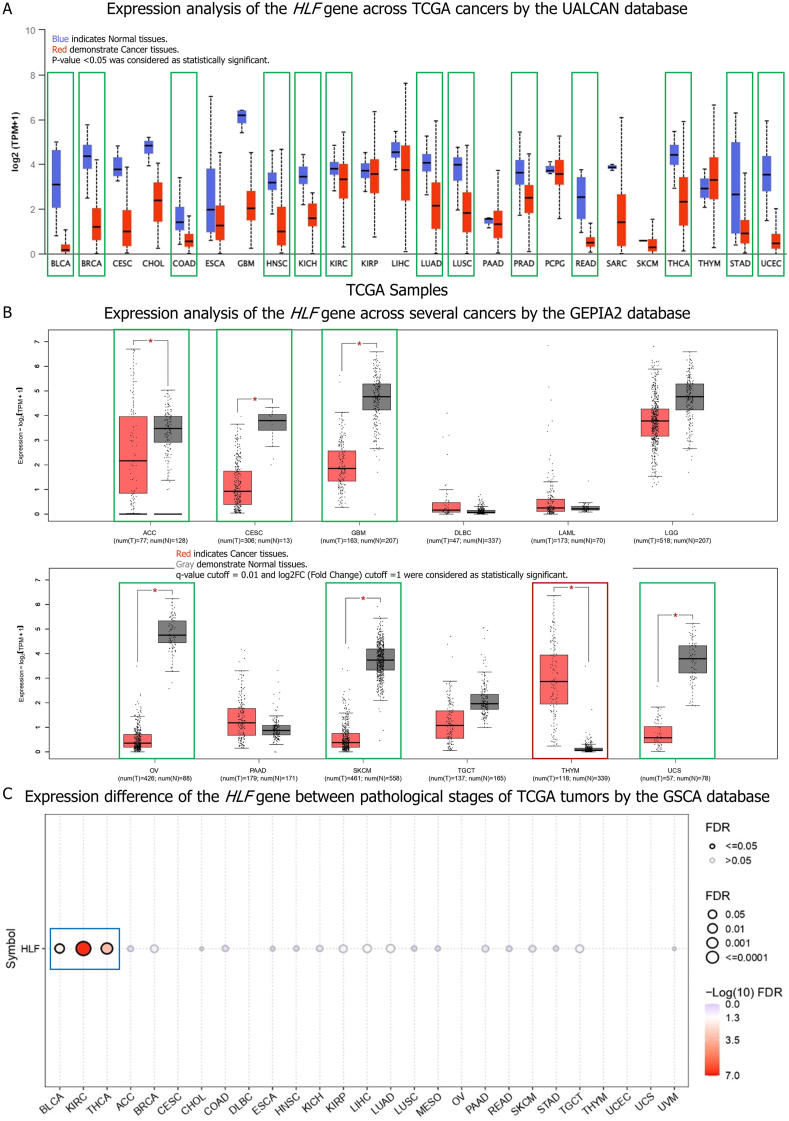
The expression analysis of the *HLF* gene and its correlation with pathological stages. Analysis of *HLF* expression across TCGA cancers in the UALCAN database; Tumors showing significant reductions are represented by green rectangles. (A). Analysis the expression of *HLF* in ACC, CESC, GBM, DLBC, LAML, LGG, OV, PAAD, SKCM, TGCT, THYM, and UCS between TCGA tumor tissues and related normal tissues in the TCGA and GTEx databases by the GEPIA2 database; Cancers that display remarkable decreases are indicated by green rectangles, whereas those demonstrating substantial increases are labeled with red rectangles. (B). Analysis of the *HLF* gene expression variation among pathological stages of TCGA tumors via the GSCA database; Cancers with notable changes in cancer pathological stages are indicated with blue rectangles (C). (For interpretation of the references to color in this figure legend, the reader is referred to the Web version of this article.)

According to the UALCAN database, the expression level of the *HLF* gene was lower in tumor tissues of BLCA, BRCA, COAD, HNSC, KIRC, KICH, LUAD, LUSC, PRAD, READ, STAD, THCA, and UCEC than in matching control tissues (Fig. 1A). The GEPIA2 database also revealed that whereas the expression of the *HLF* gene was dramatically downregulated in malignant tissues of ACC, CESC, GBM, OV, SKCM, and UCS, it was significantly upregulated in tumor tissues of patients with THYM compared to normal samples (Fig. 1B). The expression of this gene was lower in ACC, BLCA, BRCA, CESC, COAD, GBM, HNSC, KIRC, KICH, LUAD, LUSC, PRAD, READ, SKCM, STAD, THCA, UCEC, and UCS tumor tissues and greater in THYM tumor tissues. When we compared the *HLF* gene expression across pathological stages of TCGA tumors, we found a substantial difference in expression of the *HLF* gene between pathological stages of KIRC, THCA, and BLCA, with a considerable decrease observed at advanced stages (Fig. 1C).

### 3.2 Survival analysis

**Fig. 2:**
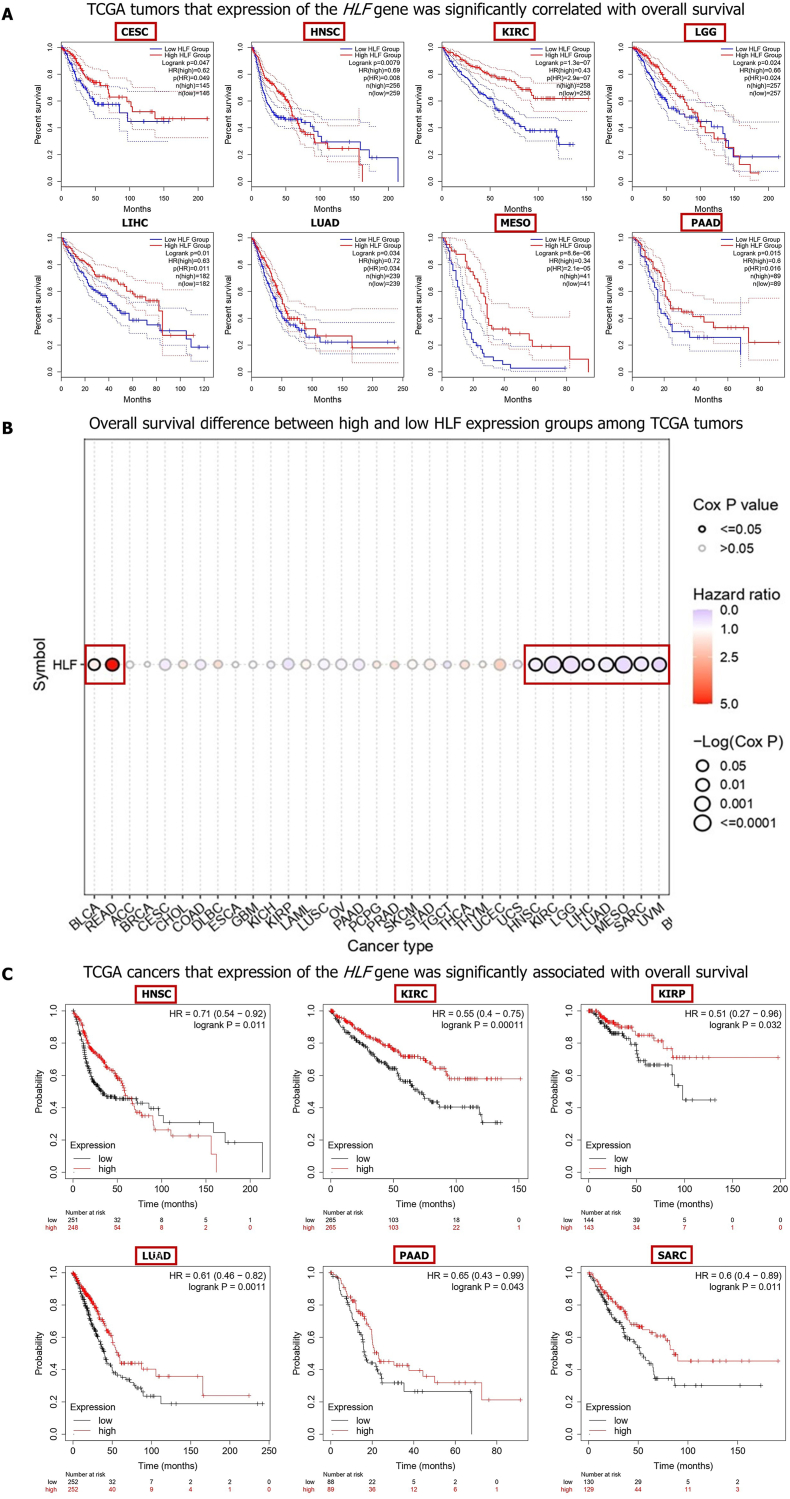
The correlation analysis between the expression of the *HLF* gene and the overall survival outcome of cancers in the TCGA database using GEPIA2 (A), GSCA (B), and Kaplan-Meier plotter (C) databases. The survival map of significant results was shown and tumors showing significant overall survival (OS) outcomes in relation to *HLF* expression were indicated by red rectangles. (For interpretation of the references to color in this figure legend, the reader is referred to the Web version of this article.)

The survival analysis using the GEPIA2 database disclosed that CESC (*P* = 0.047), HNSC (*P* = 0.0079), KIRC (*P* = 1.3e−07), LGG (*P* = 0.024), LIHC (*P* = 0.01), LUAD (*P* = 0.034), MESO (*P* = 8.6e−06), PAAD (*P* = 0.015) patients with reduced expression of the *HLF* gene experienced higher risk of death (Fig. 2A). The GSCA database also uncovered that the higher expression of the *HLF* gene was related with unfavorable OS of patients with BLCA (*P* < 0.0361) and READ (*P* < 0.0053). Besides, HNSC (*P* < 0.0098), KIRC (*P* < 0.0000), LGG (*P* < 0.0001), LIHC (*P* < 0.0337), LUAD (*P* < 0.0020), MESO (*P* < 0.0000), SARC (*P* < 0.0039), and UVM (*P* < 0.0019) patients with lower expression of the *HLF* genes had higher risk of Death (Fig. 2B; Table S3). The KM-plotter database also demonstrated that higher expression of the *HLF* gene was associated with better OS of HNSC (*P* = 0.011), KIRC (*P* = 0.0001), KIRP (*P* = 0.0324), LUAD (*P* = 0.0011), PAAD (*P* = 0.0426), and SARC (*P* = 0.0114) (Fig. 2C). In sum, these data unveil that *HLF* downregulation was correlated with better prognosis of BLCA and READ patients and poor OS outcome of patients with CESC, HNSC, KIRC, KIRP, LGG, LIHC, LUAD, MESO, PAAD, SARC, and UVM.

### 3.3 Genetic alterations of the *HLF* gene

**Fig. 3:**
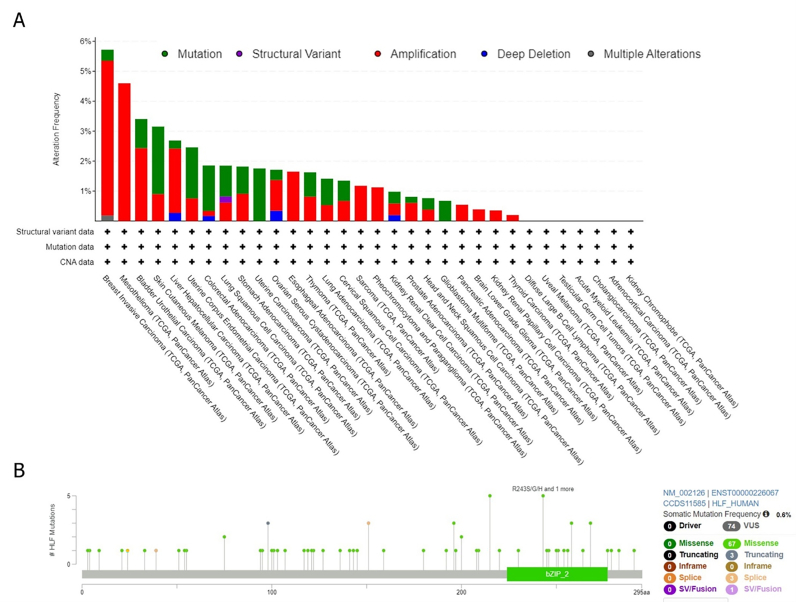
Analysis of the *HLF* gene genetic changes among TCGA cancers based on the data retrieved from the cBioPortal database. The alteration frequency with mutation type (A). The alteration frequency with mutation site (B).

The cBioPortal platform was employed to assess genetic aberrations of the *HLF* gene among diverse TCGA tumors. We observed that the *HLF* gene mutations was the most frequent in BRCA (mutated in 5.72 % of samples), followed by MESO (4.6 %) and BLCA (3.41 %). In BRCA, 5.17 %, 0.37 %, and 0.18 % of samples had amplification, mutations, and multiple alterations. All MESO, ESCA, PCPG, PAAD, LGG, KIRP, and THCA samples had only one sort of alteration: amplification (Fig. 3B). Further analyses also suggested that 0.6 % of samples had somatic mutations in the *HLF* gene. We found 74 VUS mutations and no driver mutation among them. In the case of VUS, missense (n = 67), truncating (n = 3), splice site (n = 3), and SV/fusion (n = 1) were the most types of alterations in the *HLF* gene (Fig. 3C).

### 3.4 Methylation analysis

When we used the DNMIVD database to investigate the methylation pattern of the *HLF* gene promoter, we discovered that, as shown in Table S4, between 22 different kinds of cancers, the promoter region of the *HLF* gene was significantly hypermethylated in BLCA (Beta difference = 0.175 and adjusted p-value = 2.49e-04), COAD (Beta difference = 0.111 and adjusted p-value = 1.16e-04), and PRAD (Beta difference = 0.200 and adjusted p-value <0.0000). The results of the correlation study likewise indicated a substantial inverse relationship between the amount of methylation in the promoter area and the amount of expression of the *HLF* gene in BLCA, COAD, and PRAD, showing that expression of *HLF* is mainly controlled by promoter methylation (Table S5).

### 3.5 PPI network of the *HLF* gene

**Fig. 4:**
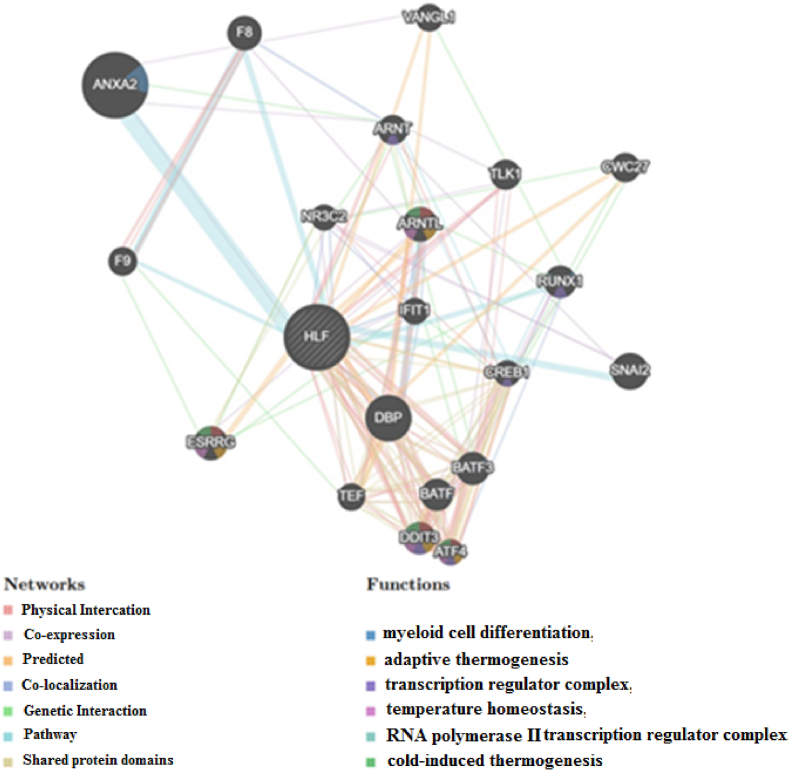
Protein-protein interaction (PPI) network and functional analysis of the *HLF* gene based on the data retrieved from the GeneMANIA database.

We applied the GeneMANIA tool to produce a PPI network for the *HLF* gene to demonstrate the probable processes that mediate cancer progression. The results demonstrated that the *HLF* gene had significant interactions with ANXA2, DBP, and SNAI2, as displayed in Fig. 4. The functional analysis revealed that the *HLF* gene and its related proteins probably participate in myeloid cell differentiation, transcription regulator complex, cold-induced thermogenesis, adaptive thermogenesis, RNA polymerase II transcription regulator complex, temperature homeostasis, regulation of cold-induced thermogenesis, and myeloid leukocyte differentiation.

### 3.6 Pathway enrichment analysis

**Fig. 5:**
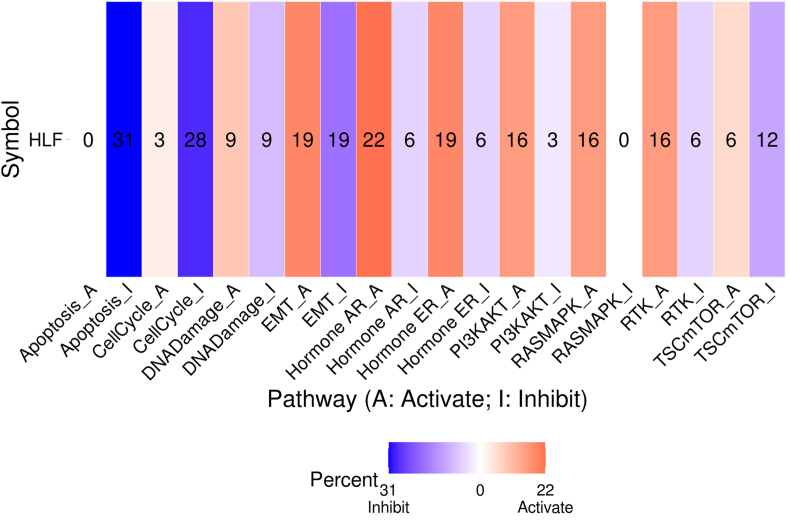
Pathway analysis of the *HLF* gene according to data obtained from the GSCA database. The number in each cell demonstrates the percentage of tumor types in which the *HLF* gene showed a significant correlation with a specific pathway among all TCGA tumors.

The GSCA database revealed that the *HLF* gene probably inhibits apoptosis (33 %), cell cycle (28 %), EMT (19 %), and activates Hormone AR (22 %), Hormone ER (19 %), EMT (19 %), PI3K/AKT (16 %), Ras/MAPK (16 %), and RTK (16 %) pathways (Fig. 5).

### 3.7 Relationship between immune cell infiltration and *HLF*

We investigated the changes in immune cell infiltration that occurred in samples that showed differences in *HLF* expression in order to determine whether or not *HLF* expression was connected with the microenvironment of the tumor in a number of different types of cancers. The expression of the *HLF* gene was shown to be related to the infiltration levels of resting Memory CD4^+^ T cell and activated Mast cell in 24 tumor types, Monocytes in 20 tumor types, Naive B cell and M2 Macrophages in 19 tumor types, Tregs, M1 Macrophages, and resting Mast cell in 16 human cancers, CD8^+^ T cells and Plasma B cell in 15 human cancers, M0 Macrophages in 12 human cancers, Memory B cell, activated NK cell, resting NK cell, and Follicular helper T cell in 10 human cancers, resting Myeloid DC in 9 human cancers, Neutrophils in 8 human cancers, activated Memory CD4^+^ T cell in 7 human cancers, Naive CD4^+^ T cell in 4 human cancers, activated Myeloid DC in 3 human cancers. Furthermore, the results also unveiled that among studied cancers, expression of the *HLF* gene was strongly associated with immune infiltration in THCA (Table S6).

### 3.8 Correlation between the *HLF* expression and drug sensitivity

**Fig. 6:**
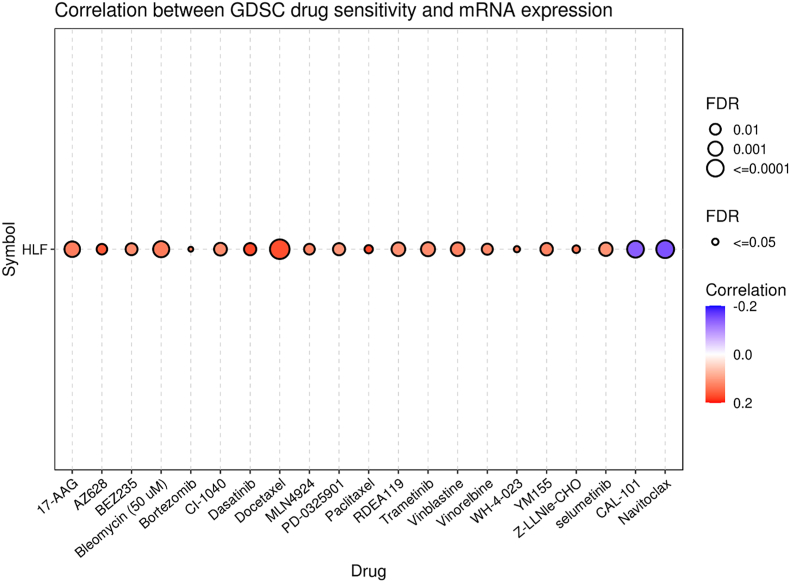
Analysis of the correlation between drug sensitivity and expression of the *HLF* gene using data from the GSCA database.

By using the GDSC IC50 drug data from the GSCA database, we investigated whether or not there was a correlation between the amount of *HLF* gene expression and how sensitive a patient was to a certain medicine. According to the findings, cancer patients with an overexpression of the *HLF* gene may be resistant to CAL-101 and Navitoclax but responsive to Paclitaxel, Dasatinib, Docetaxel, AZ628, *Z*-LLNle-CHO, WH-4-023, Bortezomib, Bleomycin (50 μM), 17-AAG, MLN4924, Vinblastine, YM155, Vinorelbine, CI-1040 (Fig. 6).

## 4 Discussion

In the first part of this paper, we conducted research on the amount of *HLF* gene expression found in human cancer. We observed a decreased expression of the *HLF* gene in the tumor tissues of many different human malignancies, such as ACC, BLCA, BRCA, CESC, COAD, GBM, HNSC, KIRC, KICH, LUAD, LUSC, PRAD, READ, SKCM, STAD, THCA, UCEC, and UCS. In addition to this, we discovered that the expression of the *HLF* gene was significantly elevated in THYM tumor tissues in comparison to normal tissue samples. In addition, we uncovered a substantial distinction in the manner in which the *HLF* gene is expressed during the pathological stages of KIRC, THCA, and BLCA. The research done by Xiang and colleagues shows that the *HLF* gene is significantly overexpressed at both the transcriptional and protein levels in HCC tissues as compared to normal tissues. In addition to this, they observed that the *HLF* expression levels in recurrent HCCs samples were noticeably higher than those of initial HCCs [20]. In a separate investigation, Hengyu Li et al. found that TNBC samples had a greater expression of the *HLF* gene [22]. In addition, Wang and colleagues found that the expression of the *HLF* gene was much reduced in lung cancer tissues when compared to normal samples [18]. Furthermore, Chen et al. have determined that the mRNA level of the *HLF* gene was apparently reduced in early-relapsed NSCLC tissues [21]. Then, we investigated the effect of the abnormal expression of the *HLF* gene on the OS outcome of cancer patients. Our findings disclosed that downregulation of the *HLF* gene was significantly associated with unfavorable OS outcomes in patients with CESC, HNSC, KIRC, KIRP, LGG, LIHC, LUAD, MESO, PAAD, SARC, and UVM, and good OS outcomes of BLCA and READ patients. Consistent with us, it has been suggested that downregulation of the *HLF* gene also was correlated with worse survival in LUAD [18]. Furthermore, another research has proposed that upregulation of the *HLF* gene may lead to a good prognosis in patients with RCC [23]. Besides, others have demonstrated that downregulation of the *HLF* gene act as a prognostic biomarker for patients with NSCLC [21]. In contrast, high *HLF* levels were related to poor DFS and OS in HCC patients [20]. These findings may indicate that the aberrant expression of the *HLF* gene in tumor tissues plays essential role in the progression of cervical, head and neck, renal, glioma, liver, lung, prostate, sarcoma, skin, and rectal cancers and probably acts as tumor suppressor gene except for liver cancer. Several mechanisms have been recognized for dysregulation of a specific gene, such as gene alterations and abnormal promoter region methylation [[33], [34], [35], [36]]. Therefore, we next explored the genetic alteration of the *HLF* gene and discovered that the *HLF* gene alterations was the most frequent in BRCA, MESO, and BLCA. We also found that missense, truncating, splice site, and SV/fusion mutations were the main aberrations in the *HLF* gene. Huang et al. have discovered that the rs6504958 polymorphism in the *HLF* gene was linked to a higher incidence of RCC. In contrast, no variation was significantly associated with the survival outcome of these patients. They also discovered that the rs6504958 G allele was connected to reduced *HLF* gene expression, which was linked to more advanced RCC [23]. Moreover, The TCF3-HLF fusion gene resulting from the t(17; 19)(q22; p13) translocation has been reported in nearly 1 % of childhood B-lineage ALL [37]. The *E2A*- *HLF* fusion gene has been documented in a limited proportion of pro-B cell ALL patients [16,38]. Chen et al. have introduced genetic deletion and methylation as a possible cause of reduced *HLF* gene expression in NSCLC patients [21]. Concerning the methylation status of the *HLF* gene promoter region, we found that there was a strong negative correlation between the hypermethylation of this area and the downregulation of the *HLF* gene in BLCA, COAD, and PRAD. These data may imply that abnormalities in the methylation level of the promoter region should not be considered as the etiology for reported variations in the expression of the *HLF* gene in the majority of malignancies, with the exception of these three. On the other hand, Kachroo et al. have shown evidence of hypomethylation in 78 % of the 7000 differentially methylated CpG sites in the TCF3-*HLF* subtype across ALL subtypes [39]. To shed light on mechanisms in which abnormal expression of the *HLF* gene participates in cancer progression and development, we first investigated the protein partners of the *HLF* gene. Next, we investigated whether or not there was a connection between the activity of a certain pathway and the expression of the *HLF* gene in human tumors. According to the findings, the *HLF* gene had substantial interactions with 19 different proteins, including ANXA2, DBP, and SNAI2. These proteins are primarily involved in the processes of myeloid cell differentiation, transcription regulator complex, cold-induced thermogenesis, adaptive thermogenesis, RNA polymerase II transcription regulator complex, temperature homeostasis, regulation of cold-induced thermogenesis, and myeloid leukocyte differentiation. It has been established that abnormal regulation of ANXA2, which is a protein that binds phospholipids and is dependent on calcium, is linked to a number of different types of cancer. It has been hypothesized that ANXA2 is overexpressed in a number of human malignancies and has a major effect on the adhesion, proliferation, apoptosis, invasion, and metastasis of tumor cells [40]. The transcription factor SNAI2 coordinates biological mechanisms essential to tissue development and tumorigenesis. It has been defined as a component of EMT, which exerts its roles in various biological mechanisms, including tumor metastasis, cellular differentiation, and DNA damage repair [41]. The findings also shown that there is a substantial correlation between the *HLF* gene and the apoptosis, cell cycle, EMT, hormone AR and hormone ER pathways, as well as the PI3K/AKT route, Ras/MAPK pathway, and RTK pathway. It has been known for a long time that any abnormalities in the natural process of cell death known as apoptosis might increase a person's risk of developing cancer [[42], [43], [44], [45], [46], [47], [48], [49]]. For example, it has been uncovered that androgen receptor activity is correlated with poor prognosis in Glioblastoma [50]. Others have reported that the Ras/MAPK pathway activation occurs in 50–100 % of hepatocellular carcinoma, associated with worse survival [51]. It has demonstrated that *HLF* enhances tumor-initiating cell generation and TIC-like properties of hepatoma cells through activation of c-Jun [20]. In TNBC, activation of gamma-glutamyltransferase 1 by HLF enhances proliferation, metastasis, and cisplatin resistance, as well as ferroptosis resistance [22]. Furthermore, it has been demonstrated that reduced *HLF* expression promotes NSCLC metastasis by modulating the PPAR/NF-b signaling pathway [21]. Besides, it has been unearthed that HLF-mediated downregulation of miR-132 leads to TTK overexpression, which contribute to proliferation, and metastasis in glioma cancerous cells [19]. Based on these observations, it has been hypothesized that abnormal regulation of the *HLF* gene might have a role in the progression and development of human malignancies via the aforementioned pathways. However, tests in both vitro and in vivo were required to be carried out in order to determine the specific role that the *HLF* gene plays in each of these pathways. Both stromal components and tumor cells contribute to the formation of the microenvironment that surrounds a tumor. It has the potential to have a significant impact on the processes of carcinogenesis, invasion, and metastasis in a wide variety of cancer cell types [[52], [53], [54]]. Immunotherapy is a relatively recent discovery that targets the immune microenvironment of tumors in order to stop the growth of cancer and prevent its spread. As a result, it is an essential component in the administration of care to cancer patients [55]. The clinical importance of immune cell infiltration has been confirmed in many cancer types, such as pancreatic cancer [56], gallbladder cancer [57], breast cancer [58], clear cell renal carcinoma [59], prostate cancer [60], and small cell lung cancer [61]. Drug resistance is a major challenge in treating human cancers [62]. Our research found a strong correlation between aberrant expression of the *HLF* gene and infiltration levels of several immune cells like CD8^+^ T cell, CD4^+^ T cell, and Tregs. Therefore, we can suggest that abnormal *HLF* expression may alter tumor immunity in a variety of human cancers and affect the successful treatment of cancer patients. Nonetheless, further research is required to determine the molecular mechanism through which *HLF* performs these effects. Hence, numerous studies have probed the roles of different genes and pathways in drug resistance. For instance, Jiang et al. have identified that the ADH1C/MAT1A axis probably promotes cisplatin resistance in lung cancer [63]. Several reports have highlighted the role of miR-34 as a crucial tumor suppressor miRNA in drug resistance in different tumors [64]. In this research, we examined the association of *HLF* expression with drug response. We realized that cancer patients with higher expression of the *HLF* gene in their tumor tissues might experience sensitivity to Paclitaxel, Dasatinib, Docetaxel, AZ628, *Z*-LLNle-CHO, WH-4-023, Bortezomib, Bleomycin (50 μM), 17-AAG, MLN4924, Vinblastine, YM155, Vinorelbine, CI-1040, BEZ235, Trametinib, RDEA119, selumetinib, and PD-0325901 and resistance to CAL-101 and Navitoclax. Paclitaxel, Dasatinib, Docetaxel, Bortezomib, and Navitoclax are renowned anti-tumor drugs with a specific mechanism of action and have considerable capacity for use in treatments of diverse cancers [[65], [66], [67], [68], [69]]. Integrating these data and the observed downregulation of the *HLF* gene in the majority of human cancer in our research, we can suggest that cancer patients with lower expression of the *HLF* gene possibly are resistant to Paclitaxel, Dasatinib, Docetaxel, AZ628, *Z*-LLNle-CHO, WH-4-023, Bortezomib, Bleomycin (50 μM), 17-AAG, MLN4924, Vinblastine, YM155, Vinorelbine, CI-1040, BEZ235, Trametinib, RDEA119, selumetinib, and PD-0325901 and sensitive to CAL-101 and Navitoclax. Therefore, analyzing the expression profile of the *HLF* gene along with other markers could be utilized as a useful predictive marker for the evaluation of cancer treatment efficacy.

It is worthy to note the limitations of our research. First, the few numbers of tissues from distinctive cancers may have led to erroneous findings. Second, we only relied on in-silico analyses; therefore, both in vivo and in vitro experiments are crucial to elucidate the specific mechanism of action of the *HLF* gene in progression and development of human cancers.

## 5 Conclusion

Our findings demonstrated that *HLF* expression was downregulated in various human cancers and this dysregulation was associated with overall survival, implying that *HLF* may be a prognostic biomarker for certain malignancies. Furthermore, we determined the potential molecular mechanisms through which *HLF* may mediate immune infiltration, cell apoptosis, EMT, and cell cycle pathways and participle in cancer progression and drug resistance. Further works are necessary to verify the potential use of *HLF* in the diagnosis, prognosis, and targeted therapy of cancers. Besides, in view of the main cellular pathways that *HLF* may inhibit, we suggest conduction of expression assays of this gene a large numbers of clinical samples.

## Ethical approval

This research was approved by the ethics committee of Hormozgan University of Medical Science (Ethical Number: IR.HUMS.REC.1400.062).

## Funding

This work has been supported by a grant (Grant Number: 990672) from the office of the Vice-Chancellor for Research, the 10.13039/501100011917Hormozgan University of Medical Sciences, Bandar Abbas.

## CRediT authorship contribution statement

**Mohsen Ahmadi:** Writing – original draft, Data curation, Conceptualization. **Amirhossein Mohajeri Khorasani:** Data curation. **Firouzeh Morshedzadeh:** Data curation. **Negin Saffarzadeh:** Formal analysis. **Sayyed Mohammad Hossein Ghaderian:** Supervision. **Soudeh Ghafouri-Fard:** Writing – review & editing. **Pegah Mousavi:** Supervision.

## Declaration of competing interest

The authors declare that they have no known competing financial interests or personal relationships that could have appeared to influence the work reported in this paper.

## Data Availability

No data was used for the research described in the article.

## Abbreviations:

ALL: Acute Lymphoblastic Leukemia
AR: Androgen Receptor
cBioPortal: cBio Cancer Genomics Portal
DFS: Disease-Free Survival
DNMIVD: DNA Methylation Interactive Visualization Database
EMT: Epithelial-Mesenchymal Transition
ER: Estrogen Receptor
FDR: False Discovery Rate
GDSC: Genomics of Drug Sensitivity in Cancer
GEPIA2: Gene expression profiling interactive analysis, version 2
GSCA: Gene Set Cancer Analysis
GTEx: Genotype-tissue expression
HCC: Hepatocellular Carcinoma
HLF: Hepatic Leukemia Factor
NSCLC: Non-Small Cell Lung Cancer
OS: Overall survival
PAS: Pathway Activity Score
PPI: Protein-Protein Interaction
RCC: Renal Cell Carcinoma
TCGA: The Cancer Genome Atlas
TIMER2: Tumor IMmune Estimation Resource, version 2
TNBC: Triple-Negative Breast Cancer
UALCAN: The University of ALabama at Birmingham CANcer data analysis Portal
VUS: Variants of Uncertain Significance
