# Supplementary material for: HLF is a promising prognostic, immunological, and therapeutic biomarker in human tumors
Source: Biochem Biophys Rep. 2024 May 1;38:101725. doi: 10.1016/j.bbrep.2024.101725 (PMC11070826; doi:10.1016/j.bbrep.2024.101725)
Supplement: Multimedia component 1 [file mmc1.docx]

**Table S1.** abbreviation and sample size for 33 cancer types based on the GEPIA2 database.

| TCGA | Detail | Tumor sample count | Normal sample count | GTEx | sample count |
| --- | --- | --- | --- | --- | --- |
| ACC | Adrenocortical carcinoma | 77 | - | Adrenal Gland | 128 |
| BLCA | Bladder Urothelial Carcinoma | 404 | 19 | Bladder | 9 |
| BRCA | Breast invasive carcinoma | 1085 | 112 | Breast | 179 |
| CESC | Cervical squamous cell carcinoma and endocervical adenocarcinoma | 306 | 3 | Cervix Uteri | 10 |
| CHOL | Cholangiocarcinoma | 36 | 9 | - | - |
| COAD | Colon adenocarcinoma | 275 | 41 | Colon | 308 |
| DLBC | Lymphoid Neoplasm Diffuse Large B-cell Lymphoma | 47 | - | Blood | 337 |
| ESCA | Esophageal carcinoma | 182 | 13 | Esophagus | 273 |
| GBM | Glioblastoma multiforme | 163 | - | Brain | 207 |
| HNSC | Head and Neck squamous cell carcinoma | 519 | 44 | - | - |
| KICH | Kidney Chromophobe | 66 | 25 | Kidney | 28 |
| KIRC | Kidney renal clear cell carcinoma | 523 | 72 | Kidney | 28 |
| KIRP | Kidney renal papillary cell carcinoma | 286 | 32 | Kidney | 28 |
| LAML | Acute Myeloid Leukemia | 173 | - | Bone Marrow | 70 |
| LGG | Brain Lower Grade Glioma | 518 | - | Brain | 207 |
| LIHC | Liver hepatocellular carcinoma | 369 | 50 | Liver | 110 |
| LUAD | Lung adenocarcinoma | 483 | 59 | Lung | 288 |
| LUSC | Lung squamous cell carcinoma | 486 | 50 | Lung | 288 |
| MESO | Mesothelioma | 87 | - | - | - |
| OV | Ovarian serous cystadenocarcinoma | 426 | - | Ovary | 88 |
| PAAD | Pancreatic adenocarcinoma | 179 | 4 | Pancreas | 167 |
| PCPG | Pheochromocytoma and Paraganglioma | 182 | 3 | - | - |
| PRAD | Prostate adenocarcinoma | 492 | 52 | Prostate | 100 |
| READ | Rectum adenocarcinoma | 92 | 10 | Colon | 308 |
| SARC | Sarcoma | 262 | 2 | - | - |
| SKCM | Skin Cutaneous Melanoma | 461 | 1 | Skin | 557 |
| STAD | Stomach adenocarcinoma | 408 | 36 | Stomach | 175 |
| TGCT | Testicular Germ Cell Tumors | 137 | - | Testis | 165 |
| THCA | Thyroid carcinoma | 512 | 59 | Thyroid | 278 |
| THYM | Thymoma | 118 | 2 | Blood | 337 |
| UCEC | Uterine Corpus Endometrial Carcinoma | 174 | 13 | Uterus | 78 |
| UCS | Uterine Carcinosarcoma | 57 | - | Uterus | 78 |
| UVM | Uveal Melanoma | 79 | - | - | - |

**Table S2.** The list of incorporated immune cells in our study.

|  | Immune cell |
| --- | --- |
| 1 | CD8+ T cell |
| 2 | activated Mast cell |
| 3 | activated Memory CD4+ T cell |
| 4 | activated Myeloid Dendritic Cell (DC) |
| 5 | activated Natural Killer (NK) cell |
| 6 | Eosinophils |
| 7 | Follicular helper T cell |
| 8 | Gamma delta T cell |
| 9 | M0 Macrophages |
| 10 | M1 Macrophages |
| 11 | M2 Macrophages |
| 12 | Memory B cell |
| 13 | Monocytes |
| 14 | Naive B cell |
| 15 | Naive CD4+ T cell |
| 16 | Neutrophils |
| 17 | Plasma B cell |
| 18 | Regulatory T cell (Tregs) |
| 19 | resting Mast cell |
| 20 | resting Memory CD4+ T cell |
| 21 | resting Myeloid DC |
| 22 | resting NK cell |

**Table S3**. The survival difference between high and low HLF expression groups among TCGA tumors by the GSCA database.

| **Cancer** | **HR_categorical(H/L)** | **Coxp_categorical** | **Log-rank p-value** | **higher_risk_of_death** |
| --- | --- | --- | --- | --- |
| ACC | 0.830543 | 0.624567 | 0.624005 | Lower expression |
| BLCA | 1.371554 | 0.036857 | 0.036072 | Higher expression |
| BRCA | 0.989983 | 0.950532 | 0.950836 | Lower expression |
| CESC | 0.640366 | 0.061587 | 0.059288 | Lower expression |
| CHOL | 1.475193 | 0.43126 | 0.4284 | Higher expression |
| COAD | 0.69395 | 0.136687 | 0.134532 | Lower expression |
| DLBC | 1.751355 | 0.444067 | 0.438198 | Higher expression |
| ESCA | 0.973385 | 0.906022 | 0.904872 | Lower expression |
| GBM | 0.913812 | 0.60309 | 0.607051 | Lower expression |
| HNSC | 0.704332 | 0.010237 | 0.009842 | Lower expression |
| KICH | 0.742479 | 0.65837 | 0.657214 | Lower expression |
| KIRC | 0.529077 | 5.32E-05 | 3.98E-05 | Lower expression |
| KIRP | 0.565028 | 0.068679 | 0.065001 | Lower expression |
| LAML | 1.250606 | 0.254049 | 0.253028 | Higher expression |
| LGG | 0.493045 | 8.51E-05 | 6.55E-05 | Lower expression |
| LIHC | 0.686084 | 0.034725 | 0.033736 | Lower expression |
| LUAD | 0.628528 | 0.002169 | 0.001974 | Lower expression |
| LUSC | 0.791696 | 0.091192 | 0.090526 | Lower expression |
| MESO | 0.371459 | 6.34E-05 | 3.49E-05 | Lower expression |
| OV | 0.789951 | 0.111641 | 0.110091 | Lower expression |
| PAAD | 0.666154 | 0.054196 | 0.052789 | Lower expression |
| PCPG | 1.454916 | 0.613615 | 0.615431 | Higher expression |
| PRAD | 1.873124 | 0.390757 | 0.383053 | Higher expression |
| READ | 4.949363 | 0.011672 | 0.005254 | Higher expression |
| SARC | 0.556392 | 0.004353 | 0.003852 | Lower expression |
| SKCM | 1.156856 | 0.281788 | 0.281855 | Higher expression |
| STAD | 1.30143 | 0.099727 | 0.098768 | Higher expression |
| TGCT | 0.538945 | 0.613828 | 0.608175 | Lower expression |
| THCA | 1.640064 | 0.328072 | 0.32326 | Higher expression |
| THYM | 1.206552 | 0.780731 | 0.780427 | Higher expression |
| UCEC | 2.005482 | 0.055831 | 0.051188 | Higher expression |
| UCS | 0.753531 | 0.411568 | 0.40958 | Lower expression |
| UVM | 0.263244 | 0.00366 | 0.001872 | Lower expression |

p-value < 0.05 was considered statistically significant.

**Table S4.** The difference between methylation level of the *HLF* gene in tumor tissues and normal samples, based on the data obtained from the DNMIVD database.

| **Cancer** | **Beta difference** | **Adjusted P-value** | **Significant** |
| --- | --- | --- | --- |
| BLCA | 0.175 | 2.49E-04 | Yes |
| BRCA | 0.080 | 3.79E-08 | Not |
| CESC | 0.043 | 1.00E+00 | Not |
| CHOL | 0.032 | 5.77E-01 | Not |
| COAD | 0.111 | 1.16E-04 | Yes |
| ESCA | 0.060 | 4.09E-01 | Not |
| GBM | 0.006 | 1.00E+00 | Not |
| HNSC | 0.030 | 5.10E-03 | Not |
| KIRC | 0.006 | 3.82E-06 | Not |
| KIRP | 0.002 | 7.13E-01 | Not |
| LIHC | -0.003 | 2.84E-01 | Not |
| LUAD | 0.009 | 5.67E-01 | Not |
| LUSC | 0.006 | 8.85E-02 | Not |
| PAAD | 0.026 | 2.82E-01 | Not |
| PCPG | 0.001 | 9.95E-01 | Not |
| PRAD | 0.200 | 0.00E+00 | Yes |
| READ | 0.055 | 2.68E-01 | Not |
| SARC | 0.024 | 7.56E-01 | Not |
| SKCM | 0.109 | 9.99E-01 | Not |
| STAD | 0.052 | 9.99E-01 | Not |
| THCA | 0.001 | 8.65E-01 | Not |
| THYM | 0.003 | 1.00E+00 | Not |

|beta value|>0.1 and Adjusted p-value < 0.05 was considered statistically significant.

**Table S5.** The correlation between methylation level of the promoter region of the HLF gene and its expression in tumor samples across TCGA cancers, based on the data retrieved from the DNMIVD database.

| **Cancer** | **Pearson rho value** | **Pearson p-value** | **Spearman rho value** | **Spearman p-value** |
| --- | --- | --- | --- | --- |
| BLCA | -0.236 | 8.696e-7 | -0.243 | 4.177e-7 |
| BRCA | -0.274 | 1.97152e-16 | -0.356 | 2.66418e-27 |
| CESC | -0.232 | 3.76E-05 | -0.353 | 2e-10 |
| CHOL | -0.327 | 2.81E-02 | -0.477 | 9.14E-04 |
| COAD | -0.277 | 5.806e-7 | -0.211 | 1.57E-04 |
| ESCA | -0.314 | 2.93E-05 | -0.356 | 1.81E-06 |
| GBM | 0.054 | 6.72E-01 | -0.193 | 1.26E-01 |
| HNSC | -0.323 | 0.00E+00 | -0.439 | 4.73823e-26 |
| KIRC | -0.327 | 6e-10 | -0.271 | 3.508e-7 |
| KIRP | -0.344 | 1.2e-9 | -0.382 | 0.00E+00 |
| LIHC | -0.247 | 3.522e-7 | -0.297 | 7e-10 |
| LUAD | -0.278 | 7e-10 | -0.225 | 7.111e-7 |
| LUSC | -0.184 | 3.37E-04 | -0.179 | 4.81E-04 |
| PAAD | -0.128 | 8.57E-02 | -0.142 | 5.67E-02 |
| PCPG | -0.093 | 2.06E-01 | -0.311 | 1.64E-05 |
| PRAD | -0.459 | 4.504e-29 | -0.477 | 1.63707e-31 |
| READ | -0.036 | 7.24E-01 | -0.073 | 4.70E-01 |
| SARC | -0.329 | 4.54e-8 | -0.467 | 0.00E+00 |
| SKCM | -0.037 | 4.28E-01 | 0.013 | 7.74E-01 |
| STAD | -0.166 | 2.26E-03 | -0.147 | 6.74E-03 |
| THCA | -0.180 | 1.78E-05 | -0.262 | 3e-10 |
| THYM | -0.096 | 2.97E-01 | -0.300 | 8.16E-04 |

rho value>0.1 and p-value < 0.05 was considered statistically significant.

**Table S6.** Correlation between expression of the *HLF* gene and infiltration level of immune cells across TCGA tumors utilizing the TIMER2.0 database.

| **Cancer** | **Immune cells** | **Rho value** | **p-value** | **Adjusted p-value** |
| --- | --- | --- | --- | --- |
| BLCA (n=408) | T cell follicular helper | 0.210312 | 4.78E-05 | 0.000478 |
| BLCA (n=408) | Mast cell activated | 0.419 | 4.46E-17 | 2.23E-15 |
| BLCA (n=408) | Mast cell resting | -0.26351 | 2.92E-07 | 1.88E-06 |
| BLCA (n=408) | NK cell activated | 0.213525 | 3.63E-05 | 0.000387 |
| BLCA (n=408) | NK cell resting | -0.1632 | 0.001683 | 0.010988 |
| BLCA (n=408) | Macrophage M1 | 0.202195 | 9.38E-05 | 0.000517 |
| BLCA (n=408) | Macrophage M2 | 0.254083 | 7.86E-07 | 7.14E-06 |
| BLCA (n=408) | Monocyte | 0.151229 | 0.003638 | 0.014194 |
| BLCA (n=408) | B cell naive | 0.306928 | 1.81E-09 | 2.79E-08 |
| BLCA (n=408) | B cell plasma | 0.183804 | 0.000394 | 0.001831 |
| BLCA (n=408) | T cell regulatory (Tregs) | 0.134277 | 0.009914 | 0.031725 |
| BLCA (n=408) | T cell CD4+ memory resting | 0.306074 | 2.02E-09 | 3.11E-08 |
| BLCA (n=408) | T cell CD4+ naive | -0.1973 | 0.000139 | 0.00082 |
| BLCA (n=408) | T cell CD8+ | 0.181971 | 0.000451 | 0.002864 |
| BRCA (n=1100) | Mast cell activated | 0.244163 | 5.87E-15 | 1.68E-13 |
| BRCA (n=1100) | Mast cell resting | -0.08212 | 0.009596 | 0.021564 |
| BRCA (n=1100) | NK cell resting | -0.0836 | 0.008362 | 0.037688 |
| BRCA (n=1100) | Macrophage M0 | -0.13653 | 1.57E-05 | 0.000103 |
| BRCA (n=1100) | Monocyte | 0.088566 | 0.005202 | 0.019344 |
| BRCA (n=1100) | Neutrophil | -0.10982 | 0.000523 | 0.004035 |
| BRCA (n=1100) | B cell naive | 0.253136 | 5.36E-16 | 2.01E-14 |
| BRCA (n=1100) | B cell plasma | 0.19684 | 3.86E-10 | 6.44E-09 |
| BRCA (n=1100) | T cell regulatory (Tregs) | -0.17518 | 2.71E-08 | 2.55E-07 |
| BRCA (n=1100) | T cell CD4+ memory activated | -0.13138 | 3.25E-05 | 0.000228 |
| BRCA (n=1100) | T cell CD4+ memory resting | 0.190704 | 1.36E-09 | 2.27E-08 |
| BRCA (n=1100) | T cell CD8+ | 0.106849 | 0.00074 | 0.004356 |
| BRCA-Basal (n=191) | Mast cell activated | 0.278525 | 0.000198 | 0.000683 |
| BRCA-Basal (n=191) | Mast cell resting | -0.19945 | 0.008328 | 0.019366 |
| BRCA-Basal (n=191) | Neutrophil | -0.18827 | 0.01285 | 0.049535 |
| BRCA-Basal (n=191) | T cell CD4+ memory activated | -0.21914 | 0.003671 | 0.014363 |
| BRCA-Basal (n=191) | T cell CD4+ memory resting | 0.221318 | 0.003337 | 0.013307 |
| BRCA-Her2 (n=82) | Macrophage M1 | 0.365432 | 0.001597 | 0.005951 |
| BRCA-Her2 (n=82) | B cell naive | 0.375073 | 0.001169 | 0.004615 |
| BRCA-LumA (n=568) | Mast cell activated | 0.232399 | 9.05E-08 | 6.96E-07 |
| BRCA-LumA (n=568) | NK cell resting | -0.10853 | 0.013549 | 0.049836 |
| BRCA-LumA (n=568) | Macrophage M0 | -0.19068 | 1.27E-05 | 8.66E-05 |
| BRCA-LumA (n=568) | Macrophage M1 | 0.112059 | 0.010778 | 0.030077 |
| BRCA-LumA (n=568) | Macrophage M2 | 0.116805 | 0.007848 | 0.022747 |
| BRCA-LumA (n=568) | B cell memory | -0.11691 | 0.007794 | 0.023381 |
| BRCA-LumA (n=568) | B cell naive | 0.303668 | 1.72E-12 | 3.33E-11 |
| BRCA-LumA (n=568) | B cell plasma | 0.292275 | 1.22E-11 | 2.28E-10 |
| BRCA-LumA (n=568) | T cell regulatory (Tregs) | -0.19924 | 4.99E-06 | 2.96E-05 |
| BRCA-LumA (n=568) | T cell CD4+ memory resting | 0.257047 | 3.02E-09 | 4.53E-08 |
| BRCA-LumA (n=568) | T cell CD8+ | 0.191193 | 1.20E-05 | 0.00012 |
| BRCA-LumB (n=219) | Macrophage M1 | 0.184822 | 0.010276 | 0.029083 |
| BRCA-LumB (n=219) | B cell memory | -0.20307 | 0.004729 | 0.015505 |
| BRCA-LumB (n=219) | B cell naive | 0.222252 | 0.001946 | 0.007344 |
| BRCA-LumB (n=219) | B cell plasma | 0.171613 | 0.017308 | 0.045748 |
| CESC (n=306) | T cell follicular helper | 0.20215 | 0.000714 | 0.005191 |
| CESC (n=306) | Mast cell activated | 0.222859 | 0.000184 | 0.000647 |
| CESC (n=306) | Mast cell resting | -0.22607 | 0.000148 | 0.000547 |
| CESC (n=306) | Myeloid dendritic cell resting | 0.356873 | 9.64E-10 | 1.07E-08 |
| CESC (n=306) | Myeloid dendritic cell resting | 0.356873 | 9.64E-10 | 1.07E-08 |
| CESC (n=306) | Macrophage M1 | 0.248396 | 2.90E-05 | 0.000183 |
| CESC (n=306) | Macrophage M2 | 0.217101 | 0.000272 | 0.001296 |
| CESC (n=306) | Neutrophil | -0.17235 | 0.004014 | 0.019185 |
| CESC (n=306) | B cell memory | 0.146365 | 0.014763 | 0.040082 |
| CESC (n=306) | T cell CD4+ memory resting | 0.193332 | 0.001222 | 0.005619 |
| CESC (n=306) | T cell CD8+ | 0.153357 | 0.01059 | 0.038161 |
| CHOL (n=36) | Monocyte | 0.49901 | 0.002277 | 0.009679 |
| COAD (n=458) | Mast cell activated | 0.217735 | 0.000275 | 0.000916 |
| COAD (n=458) | Macrophage M0 | 0.252536 | 2.26E-05 | 0.000146 |
| COAD (n=458) | Macrophage M1 | 0.204909 | 0.000629 | 0.002657 |
| COAD (n=458) | Macrophage M2 | 0.267978 | 6.59E-06 | 4.95E-05 |
| COAD (n=458) | B cell naive | 0.229395 | 0.000124 | 0.00067 |
| COAD (n=458) | B cell plasma | 0.165264 | 0.006014 | 0.018891 |
| COAD (n=458) | T cell regulatory (Tregs) | 0.206789 | 0.000559 | 0.002292 |
| COAD (n=458) | T cell CD4+ memory resting | 0.211657 | 0.000409 | 0.002096 |
| COAD (n=458) | T cell CD8+ | 0.147926 | 0.014072 | 0.048523 |
| ESCA (n=185) | Mast cell activated | 0.356368 | 9.11E-07 | 4.79E-06 |
| ESCA (n=185) | Mast cell resting | -0.31474 | 1.68E-05 | 7.83E-05 |
| ESCA (n=185) | Myeloid dendritic cell resting | 0.289475 | 8.10E-05 | 0.000481 |
| ESCA (n=185) | Myeloid dendritic cell resting | 0.289475 | 8.10E-05 | 0.000481 |
| ESCA (n=185) | Macrophage M1 | 0.210346 | 0.004594 | 0.014356 |
| ESCA (n=185) | Macrophage M2 | 0.176598 | 0.017717 | 0.045624 |
| ESCA (n=185) | Monocyte | 0.24197 | 0.001066 | 0.004974 |
| ESCA (n=185) | Neutrophil | -0.2282 | 0.002062 | 0.012336 |
| ESCA (n=185) | B cell plasma | 0.225078 | 0.002383 | 0.008719 |
| GBM (n=153) | NK cell activated | 0.213132 | 0.012399 | 0.048213 |
| GBM (n=153) | B cell plasma | 0.233364 | 0.006062 | 0.018943 |
| GBM (n=153) | T cell regulatory (Tregs) | -0.29707 | 0.000423 | 0.00183 |
| GBM (n=153) | T cell CD4+ memory activated | -0.23405 | 0.00591 | 0.021443 |
| HNSC (n=522) | T cell follicular helper | 0.445306 | 2.44E-25 | 1.95E-23 |
| HNSC (n=522) | Mast cell activated | 0.326668 | 1.07E-13 | 1.94E-12 |
| HNSC (n=522) | Mast cell resting | -0.2274 | 3.43E-07 | 2.08E-06 |
| HNSC (n=522) | NK cell activated | 0.128414 | 0.004332 | 0.022724 |
| HNSC (n=522) | Myeloid dendritic cell resting | 0.173013 | 0.000115 | 0.000672 |
| HNSC (n=522) | Myeloid dendritic cell resting | 0.173013 | 0.000115 | 0.000672 |
| HNSC (n=522) | Macrophage M1 | 0.164128 | 0.000256 | 0.001239 |
| HNSC (n=522) | Macrophage M2 | 0.187799 | 2.76E-05 | 0.000176 |
| HNSC (n=522) | Monocyte | 0.14754 | 0.00103 | 0.004902 |
| HNSC (n=522) | B cell memory | 0.18486 | 3.70E-05 | 0.000229 |
| HNSC (n=522) | B cell naive | 0.234733 | 1.39E-07 | 1.41E-06 |
| HNSC (n=522) | B cell plasma | 0.406656 | 5.12E-21 | 2.79E-19 |
| HNSC (n=522) | T cell regulatory (Tregs) | 0.388107 | 3.91E-19 | 2.08E-17 |
| HNSC (n=522) | T cell CD4+ memory resting | 0.227093 | 3.56E-07 | 3.73E-06 |
| HNSC (n=522) | T cell CD4+ naive | -0.11994 | 0.007739 | 0.026262 |
| HNSC (n=522) | T cell CD8+ | 0.332775 | 3.47E-14 | 2.31E-12 |
| HNSC-HPV- (n=422) | T cell follicular helper | 0.319016 | 6.50E-11 | 2.60E-09 |
| HNSC-HPV- (n=422) | Mast cell activated | 0.330176 | 1.25E-11 | 1.93E-10 |
| HNSC-HPV- (n=422) | Mast cell resting | -0.21844 | 1.04E-05 | 4.96E-05 |
| HNSC-HPV- (n=422) | Myeloid dendritic cell activated | 0.125109 | 0.012273 | 0.040369 |
| HNSC-HPV- (n=422) | Myeloid dendritic cell resting | 0.144055 | 0.003887 | 0.015169 |
| HNSC-HPV- (n=422) | Myeloid dendritic cell activated | 0.125109 | 0.012273 | 0.040369 |
| HNSC-HPV- (n=422) | Myeloid dendritic cell resting | 0.144055 | 0.003887 | 0.015169 |
| HNSC-HPV- (n=422) | Macrophage M2 | 0.166461 | 0.000831 | 0.003348 |
| HNSC-HPV- (n=422) | Monocyte | 0.122391 | 0.014311 | 0.046555 |
| HNSC-HPV- (n=422) | B cell naive | 0.177123 | 0.000371 | 0.001741 |
| HNSC-HPV- (n=422) | B cell plasma | 0.405491 | 2.91E-17 | 1.16E-15 |
| HNSC-HPV- (n=422) | T cell regulatory (Tregs) | 0.327772 | 1.80E-11 | 3.20E-10 |
| HNSC-HPV- (n=422) | T cell CD4+ memory resting | 0.218252 | 1.06E-05 | 8.65E-05 |
| HNSC-HPV- (n=422) | T cell CD8+ | 0.192079 | 0.000111 | 0.000836 |
| HNSC-HPV+ (n=98) | T cell follicular helper | 0.619065 | 1.00E-10 | 2.68E-09 |
| HNSC-HPV+ (n=98) | NK cell activated | 0.354978 | 0.000642 | 0.005133 |
| HNSC-HPV+ (n=98) | NK cell resting | -0.31624 | 0.002535 | 0.015025 |
| HNSC-HPV+ (n=98) | Macrophage M1 | 0.440483 | 1.56E-05 | 0.000103 |
| HNSC-HPV+ (n=98) | Neutrophil | -0.27618 | 0.008798 | 0.036023 |
| HNSC-HPV+ (n=98) | B cell memory | 0.30171 | 0.004061 | 0.013689 |
| HNSC-HPV+ (n=98) | B cell naive | 0.313595 | 0.002767 | 0.009882 |
| HNSC-HPV+ (n=98) | B cell plasma | 0.401691 | 9.55E-05 | 0.000526 |
| HNSC-HPV+ (n=98) | T cell regulatory (Tregs) | 0.512629 | 2.81E-07 | 2.25E-06 |
| HNSC-HPV+ (n=98) | T cell CD8+ | 0.593614 | 8.74E-10 | 2.75E-08 |
| KICH (n=66) | Monocyte | 0.379056 | 0.001847 | 0.008139 |
| KIRC (n=533) | T cell follicular helper | -0.1622 | 0.000472 | 0.003775 |
| KIRC (n=533) | Mast cell activated | 0.277136 | 1.42E-09 | 1.67E-08 |
| KIRC (n=533) | NK cell resting | 0.133438 | 0.004103 | 0.021885 |
| KIRC (n=533) | Macrophage M0 | -0.22271 | 1.37E-06 | 1.17E-05 |
| KIRC (n=533) | Macrophage M1 | 0.161999 | 0.00048 | 0.00218 |
| KIRC (n=533) | Macrophage M2 | 0.20462 | 9.49E-06 | 6.70E-05 |
| KIRC (n=533) | Monocyte | 0.242477 | 1.36E-07 | 2.09E-06 |
| KIRC (n=533) | B cell memory | -0.13303 | 0.004219 | 0.014064 |
| KIRC (n=533) | B cell naive | 0.13648 | 0.003323 | 0.011462 |
| KIRC (n=533) | T cell regulatory (Tregs) | -0.27833 | 1.20E-09 | 1.37E-08 |
| KIRC (n=533) | T cell CD4+ memory activated | -0.16404 | 0.000405 | 0.002093 |
| KIRC (n=533) | T cell CD4+ memory resting | 0.193249 | 2.95E-05 | 0.000211 |
| KIRP (n=290) | Mast cell activated | 0.300679 | 8.63E-07 | 4.67E-06 |
| KIRP (n=290) | Mast cell resting | -0.20792 | 0.000778 | 0.002256 |
| KIRP (n=290) | B cell naive | -0.17517 | 0.004775 | 0.015569 |
| LGG (n=516) | Mast cell activated | -0.16457 | 0.000302 | 0.000976 |
| LGG (n=516) | NK cell resting | -0.22537 | 6.40E-07 | 1.37E-05 |
| LGG (n=516) | Macrophage M1 | -0.14961 | 0.001035 | 0.004018 |
| LGG (n=516) | Macrophage M2 | -0.44344 | 1.91E-24 | 1.27E-22 |
| LGG (n=516) | Monocyte | -0.18128 | 6.72E-05 | 0.0004 |
| LGG (n=516) | B cell memory | -0.11883 | 0.009309 | 0.027379 |
| LGG (n=516) | B cell plasma | 0.527066 | 1.58E-35 | 1.58E-33 |
| LGG (n=516) | T cell regulatory (Tregs) | -0.28683 | 1.67E-10 | 2.67E-09 |
| LGG (n=516) | T cell CD4+ memory resting | -0.32782 | 1.94E-13 | 7.66E-12 |
| LGG (n=516) | T cell CD4+ naive | 0.210565 | 3.42E-06 | 3.04E-05 |
| LIHC (n=371) | T cell follicular helper | -0.1719 | 0.00135 | 0.006861 |
| LIHC (n=371) | NK cell resting | 0.136052 | 0.011417 | 0.04567 |
| LIHC (n=371) | Macrophage M0 | -0.13297 | 0.013445 | 0.035694 |
| LIHC (n=371) | Monocyte | 0.203202 | 0.000145 | 0.000819 |
| LIHC (n=371) | T cell regulatory (Tregs) | -0.19341 | 0.000302 | 0.001381 |
| LUAD (n=515) | Mast cell activated | 0.501497 | 9.28E-33 | 1.86E-30 |
| LUAD (n=515) | Mast cell resting | -0.2198 | 8.29E-07 | 4.60E-06 |
| LUAD (n=515) | Myeloid dendritic cell activated | 0.164903 | 0.000236 | 0.001251 |
| LUAD (n=515) | Myeloid dendritic cell resting | 0.343844 | 3.97E-15 | 9.87E-14 |
| LUAD (n=515) | Myeloid dendritic cell activated | 0.164903 | 0.000236 | 0.001251 |
| LUAD (n=515) | Myeloid dendritic cell resting | 0.343844 | 3.97E-15 | 9.87E-14 |
| LUAD (n=515) | Macrophage M0 | -0.20727 | 3.47E-06 | 2.70E-05 |
| LUAD (n=515) | Macrophage M2 | 0.307756 | 2.82E-12 | 5.47E-11 |
| LUAD (n=515) | Monocyte | 0.334319 | 2.45E-14 | 1.94E-12 |
| LUAD (n=515) | B cell memory | 0.228871 | 2.79E-07 | 2.50E-06 |
| LUAD (n=515) | T cell regulatory (Tregs) | 0.128135 | 0.004377 | 0.014591 |
| LUAD (n=515) | T cell CD4+ memory activated | -0.28876 | 6.37E-11 | 1.38E-09 |
| LUAD (n=515) | T cell CD4+ memory resting | 0.421382 | 1.22E-22 | 1.53E-20 |
| LUSC (n=501) | Mast cell activated | 0.264495 | 4.46E-09 | 4.43E-08 |
| LUSC (n=501) | Mast cell resting | -0.18741 | 3.81E-05 | 0.000152 |
| LUSC (n=501) | Myeloid dendritic cell resting | 0.119835 | 0.008798 | 0.030986 |
| LUSC (n=501) | Myeloid dendritic cell resting | 0.119835 | 0.008798 | 0.030986 |
| LUSC (n=501) | Macrophage M2 | 0.133301 | 0.003537 | 0.011725 |
| LUSC (n=501) | Monocyte | 0.19685 | 1.49E-05 | 0.000104 |
| LUSC (n=501) | Neutrophil | -0.1195 | 0.008991 | 0.036023 |
| LUSC (n=501) | B cell naive | 0.193921 | 2.00E-05 | 0.000129 |
| LUSC (n=501) | B cell plasma | 0.134852 | 0.003168 | 0.01118 |
| LUSC (n=501) | T cell CD4+ memory resting | 0.15388 | 0.000746 | 0.003561 |
| MESO (n=87) | T cell CD4+ memory resting | 0.30628 | 0.004361 | 0.016752 |
| OV (n=303) | Mast cell resting | -0.19371 | 0.002137 | 0.00555 |
| OV (n=303) | Monocyte | 0.154344 | 0.014774 | 0.046882 |
| PAAD (n=179) | Mast cell activated | 0.384868 | 2.02E-07 | 1.35E-06 |
| PAAD (n=179) | Myeloid dendritic cell resting | 0.20111 | 0.00835 | 0.02994 |
| PAAD (n=179) | Myeloid dendritic cell resting | 0.20111 | 0.00835 | 0.02994 |
| PAAD (n=179) | Macrophage M0 | -0.24002 | 0.001567 | 0.005876 |
| PAAD (n=179) | Macrophage M1 | 0.260807 | 0.00057 | 0.002471 |
| PAAD (n=179) | Macrophage M2 | 0.460059 | 2.44E-10 | 4.19E-09 |
| PAAD (n=179) | Monocyte | 0.355129 | 1.88E-06 | 1.79E-05 |
| PAAD (n=179) | B cell naive | 0.348516 | 2.99E-06 | 2.19E-05 |
| PAAD (n=179) | B cell plasma | 0.27032 | 0.000349 | 0.00165 |
| PAAD (n=179) | T cell CD4+ memory resting | 0.46828 | 1.06E-10 | 2.15E-09 |
| PAAD (n=179) | T cell CD8+ | 0.446463 | 9.33E-10 | 2.75E-08 |
| PRAD (n=498) | Mast cell activated | 0.312912 | 6.68E-11 | 9.55E-10 |
| PRAD (n=498) | NK cell activated | 0.121309 | 0.013289 | 0.049446 |
| PRAD (n=498) | Macrophage M2 | 0.216944 | 8.02E-06 | 5.79E-05 |
| PRAD (n=498) | Monocyte | 0.139907 | 0.004249 | 0.016312 |
| PRAD (n=498) | Neutrophil | 0.141129 | 0.003923 | 0.019185 |
| PRAD (n=498) | B cell memory | -0.12422 | 0.011217 | 0.031896 |
| PRAD (n=498) | B cell naive | 0.285904 | 2.88E-09 | 4.12E-08 |
| PRAD (n=498) | B cell plasma | 0.360289 | 3.39E-14 | 9.24E-13 |
| PRAD (n=498) | T cell regulatory (Tregs) | -0.20785 | 1.93E-05 | 0.000106 |
| PRAD (n=498) | T cell CD4+ memory resting | 0.297723 | 5.82E-10 | 1.11E-08 |
| PRAD (n=498) | T cell CD8+ | 0.144681 | 0.0031 | 0.014485 |
| SARC (n=260) | Mast cell activated | 0.364008 | 4.66E-09 | 4.43E-08 |
| SARC (n=260) | Mast cell resting | -0.26342 | 3.08E-05 | 0.000126 |
| SARC (n=260) | NK cell resting | -0.16352 | 0.010518 | 0.043265 |
| SARC (n=260) | Macrophage M0 | -0.20346 | 0.001397 | 0.005339 |
| SARC (n=260) | Monocyte | 0.189729 | 0.002925 | 0.012002 |
| SARC (n=260) | B cell naive | 0.289282 | 4.35E-06 | 3.14E-05 |
| SARC (n=260) | T cell CD4+ memory activated | -0.19676 | 0.002016 | 0.008581 |
| SARC (n=260) | T cell CD4+ memory resting | 0.251066 | 7.33E-05 | 0.000462 |
| SKCM (n=471) | T cell follicular helper | 0.149279 | 0.001372 | 0.006861 |
| SKCM (n=471) | Mast cell activated | 0.174675 | 0.000175 | 0.000625 |
| SKCM (n=471) | Mast cell resting | -0.19392 | 2.99E-05 | 0.000125 |
| SKCM (n=471) | NK cell activated | 0.148247 | 0.001483 | 0.010314 |
| SKCM (n=471) | NK cell resting | -0.12712 | 0.006505 | 0.030612 |
| SKCM (n=471) | Macrophage M0 | -0.13567 | 0.003664 | 0.012038 |
| SKCM (n=471) | Macrophage M2 | 0.164949 | 0.000399 | 0.00187 |
| SKCM (n=471) | B cell naive | 0.208766 | 6.78E-06 | 4.73E-05 |
| SKCM (n=471) | T cell CD4+ memory resting | 0.197075 | 2.20E-05 | 0.000161 |
| SKCM (n=471) | T cell CD8+ | 0.121635 | 0.009246 | 0.034891 |
| SKCM-Metastasis (n=368) | T cell follicular helper | 0.158011 | 0.002871 | 0.012089 |
| SKCM-Metastasis (n=368) | Mast cell activated | 0.170018 | 0.001323 | 0.003726 |
| SKCM-Metastasis (n=368) | Mast cell resting | -0.20489 | 0.000103 | 0.00039 |
| SKCM-Metastasis (n=368) | NK cell activated | 0.136305 | 0.010244 | 0.043265 |
| SKCM-Metastasis (n=368) | Macrophage M0 | -0.15217 | 0.004109 | 0.0134 |
| SKCM-Metastasis (n=368) | Macrophage M2 | 0.204226 | 0.000109 | 0.000589 |
| SKCM-Metastasis (n=368) | B cell naive | 0.261202 | 6.23E-07 | 5.08E-06 |
| SKCM-Metastasis (n=368) | T cell CD4+ memory resting | 0.212173 | 5.73E-05 | 0.00038 |
| SKCM-Primary (n=103) | NK cell activated | 0.288881 | 0.003233 | 0.018151 |
| SKCM-Primary (n=103) | Myeloid dendritic cell resting | 0.261653 | 0.007897 | 0.029102 |
| SKCM-Primary (n=103) | Myeloid dendritic cell resting | 0.261653 | 0.007897 | 0.029102 |
| SKCM-Primary (n=103) | Macrophage M1 | 0.276817 | 0.004855 | 0.015093 |
| SKCM-Primary (n=103) | T cell regulatory (Tregs) | 0.23838 | 0.015833 | 0.047798 |
| STAD (n=415) | Mast cell activated | 0.393508 | 1.74E-15 | 5.79E-14 |
| STAD (n=415) | Mast cell resting | -0.2594 | 3.03E-07 | 1.90E-06 |
| STAD (n=415) | Macrophage M2 | 0.287078 | 1.27E-08 | 1.52E-07 |
| STAD (n=415) | Monocyte | 0.263796 | 1.88E-07 | 2.48E-06 |
| STAD (n=415) | Neutrophil | -0.16901 | 0.000956 | 0.006924 |
| STAD (n=415) | B cell naive | 0.29129 | 7.57E-09 | 9.27E-08 |
| STAD (n=415) | T cell CD4+ memory resting | 0.334847 | 2.21E-11 | 5.36E-10 |
| STAD (n=415) | T cell CD8+ | 0.175287 | 0.000609 | 0.003633 |
| TGCT (n=150) | Mast cell activated | 0.372751 | 3.33E-06 | 1.66E-05 |
| TGCT (n=150) | Macrophage M0 | 0.23384 | 0.004364 | 0.013863 |
| TGCT (n=150) | B cell naive | 0.41547 | 1.67E-07 | 1.62E-06 |
| TGCT (n=150) | B cell plasma | 0.301198 | 0.00021 | 0.001039 |
| TGCT (n=150) | T cell regulatory (Tregs) | 0.380236 | 2.03E-06 | 1.25E-05 |
| TGCT (n=150) | T cell CD4+ memory resting | 0.532914 | 3.70E-12 | 1.06E-10 |
| TGCT (n=150) | T cell CD8+ | 0.239835 | 0.003435 | 0.015344 |
| THCA (n=509) | T cell follicular helper | -0.20556 | 4.69E-06 | 6.25E-05 |
| THCA (n=509) | Mast cell activated | -0.13665 | 0.002485 | 0.006292 |
| THCA (n=509) | Mast cell resting | 0.10335 | 0.02241 | 0.046687 |
| THCA (n=509) | NK cell activated | -0.23596 | 1.34E-07 | 3.06E-06 |
| THCA (n=509) | NK cell resting | 0.203383 | 5.93E-06 | 7.59E-05 |
| THCA (n=509) | Myeloid dendritic cell activated | -0.22066 | 8.53E-07 | 6.93E-06 |
| THCA (n=509) | Myeloid dendritic cell resting | -0.31297 | 1.50E-12 | 2.59E-11 |
| THCA (n=509) | Myeloid dendritic cell activated | -0.22066 | 8.53E-07 | 6.93E-06 |
| THCA (n=509) | Myeloid dendritic cell resting | -0.31297 | 1.50E-12 | 2.59E-11 |
| THCA (n=509) | Macrophage M0 | -0.33804 | 1.65E-14 | 4.30E-13 |
| THCA (n=509) | Macrophage M1 | -0.15551 | 0.000566 | 0.002471 |
| THCA (n=509) | Macrophage M2 | -0.33493 | 2.95E-14 | 7.08E-13 |
| THCA (n=509) | Monocyte | -0.13143 | 0.003632 | 0.014194 |
| THCA (n=509) | B cell memory | -0.26609 | 2.36E-09 | 3.54E-08 |
| THCA (n=509) | T cell regulatory (Tregs) | -0.45675 | 1.59E-26 | 2.55E-24 |
| THCA (n=509) | T cell CD4+ memory resting | -0.32148 | 3.40E-13 | 1.26E-11 |
| THCA (n=509) | T cell CD4+ naive | 0.17488 | 0.000103 | 0.000618 |
| THCA (n=509) | T cell CD8+ | -0.19123 | 2.11E-05 | 0.000188 |
| THYM (n=120) | Mast cell activated | 0.234494 | 0.011653 | 0.025896 |
| THYM (n=120) | NK cell activated | 0.346236 | 0.00015 | 0.001504 |
| THYM (n=120) | Macrophage M1 | 0.428582 | 1.77E-06 | 1.45E-05 |
| THYM (n=120) | Macrophage M2 | 0.276469 | 0.002781 | 0.009816 |
| THYM (n=120) | Monocyte | 0.4182 | 3.31E-06 | 2.92E-05 |
| THYM (n=120) | T cell CD4+ memory activated | -0.27697 | 0.00273 | 0.011024 |
| THYM (n=120) | T cell CD4+ memory resting | 0.251291 | 0.006749 | 0.023879 |
| THYM (n=120) | T cell CD8+ | 0.305703 | 0.000892 | 0.0051 |
| UCEC (n=545) | Mast cell activated | 0.29019 | 0.006096 | 0.014343 |
| UCEC (n=545) | T cell CD4+ memory resting | 0.284041 | 0.00732 | 0.0252 |
| UCS (n=57) | Macrophage M1 | 0.403423 | 0.002741 | 0.009732 |
| UCS (n=57) | Macrophage M2 | 0.446318 | 0.000809 | 0.003324 |
| UCS (n=57) | T cell CD4+ memory resting | 0.397015 | 0.003246 | 0.013026 |
| UVM (n=80) | Monocyte | 0.282065 | 0.012944 | 0.043922 |
